# Using head-mounted eye trackers to explore children’s color preferences and perceptions of toys with different color gradients

**DOI:** 10.3389/fpsyg.2023.1205213

**Published:** 2023-12-21

**Authors:** Zihe Chen, Tingmin Yan, YuXin Cai, Tianjian Cui, Shangbin Chen

**Affiliations:** ^1^School of Art, Southeast University, Nanjing, China; ^2^Weiyang College, Tsinghua University, Beijing, China; ^3^Costume Institute, Zhejiang Fashion Institute of Technology, Ningbo, Zhejiang, China

**Keywords:** child color preference, color gradients, visual attractiveness, visual comfort, color attributes

## Abstract

This study investigated how color gradients affect the attraction and visual comfort of children aged 4 to 7 years. We analyzed 108 eye-tracking datasets, including the color attraction index (COI), visual comfort index (PUI), and saccade rate (SR). The findings revealed that children are more attracted to colors as saturation decreases and brightness increases within a specific range. Beyond this range, reduced saturation diminishes color appeal. Moderate brightness and contrast enhance visual comfort during play, while extremely low contrast hinders concentration. Warm colors (red, orange, and yellow) slightly dominate preferences; however, the roles of hue, saturation, and brightness in children’s color preferences remain inconclusive. These insights have practical implications for age-appropriate toy design and marketing. Future research should explore age-specific color preferences for more targeted design strategies.

## 1 Introduction

Color preferences and perception have consistently been a research area of interest across multiple disciplines, based on the idea that individuals begin to receive various color stimuli from birth (Maule et al., 2023). Studies indicate that children’s neural networks are shaped by active and pleasurable psychological activities, and a lack of stimuli in their environments can impact children’s brain development (Sari, 2004). Furthermore, color recognition holds great significance for children aged 0 to 8 years, serving as a visual stimulus element; perceiving colors contributes to improving attention span and cognitive development (Jalil et al., 2013). Colors can create a pleasant and comfortable atmosphere for children as opposed to adults, thereby motivating them to learn, providing stimulation, and ultimately enhancing their educational development. Concerning colored toys, preschool children aged 3 to 6 years tend to categorize their preferences based on color rather than shape (Kagan and Lemkin, 1961). This pattern emphasizes the significant role of color perception in children’s overall understanding of objects, underscoring the importance of research on toy colors (Brian and Goodenough, 1929).

Numerous studies have confirmed that children tend to prefer vivid and bright colors. Research by Gyu (2014) provides further evidence of this notion by confirming a positive correlation between saturation and children’s preferences within the red, green, blue, and purple hue series. Interestingly, within the yellow hue series, brightness was positively correlated with preference. Additionally, Camgöz (2000) proposed that colors with high saturation and brightness tend to attract attention under any background, making bright and vivid colors the most preferred. Simultaneously, children exhibit age-related preferences for the color attributes of saturation and brightness (Cerrato, 2012). Typically, children tend to react positively to bright colors, while deeper shades may evoke negative emotions. As children grow older, their emotional responses to vibrant colors become increasingly positive. Furthermore, sex differences exist in color attribute preferences, which might be influenced by socio-cultural factors, such as the cultural association of pink with girls (Davis et al., 2021).

Unlike the additive color model used in computer displays (Labrecque, 2020), organic dyes and pigments in the physical world adhere to the subtractive color model and are constrained by their physical properties (Volz and Simon, 2001). Therefore, no single color simultaneously possesses the highest saturation and brightness, making the direct application of theoretically optimal colors to toy manufacturing challenging.

In the context of toy color design, increasing the brightness of the dye commonly results in a loss of saturation. When selecting vibrant and bright colors within a hue, a range of color gradients representing the highest brightness at the current level of saturation is obtained, rather than a single color. Many studies on children’s color preferences utilize screens and lighting as mediums for color (Wong and Hines, 2015; Grandgeorge and Masataka, 2016), often employing a single variable related to color attributes (hue, brightness, or chroma) in experiments (Wang and Cho, 2022). However, the discrepancy between screen lighting and dye color mixing models makes the direct application of theoretically optimal colors to product manufacturing challenging.

Toy manufacturers face a practical challenge; even though they employ theoretically high-saturation and high-brightness colors in their designs (Cerrato, 2012), this theoretical application results in a range of color gradients when applied to dyes. Consequently, the color schemes of toys on the market are not standardized but generally approximate the envisioned color gradient distribution. Within this color gradient, the predominant color schemes tend to be those achieving higher brightness without compromising saturation. However, a definitive consensus has not yet been reached on whether children favor color schemes that progressively increase in brightness while sacrificing saturation along the color gradient. Whether children’s color preferences adhere to a linear color effect remains unclear.

Research on color preferences and perception often employs both behavioral and physiological measurement methods (Palmer et al., 2013). Physiological measurements primarily focus on eye movements during screen-based eye-tracking tasks (Lee et al., 2005; Kooiker et al., 2016). However, observing children while they play with toys involves dynamic tasks of active exploration, which are often extended over a significant duration. Consequently, screen-based eye-tracking physiological measurements have some limitations within the scope of this research.

First, these methods have primarily been conducted in short-duration experiments and focused on the immediate effects of color stimuli. With increasing emphasis on visual quality, visual comfort is considered a significant factor influencing color perception (Zhang et al., 2022), which is often objectively measured through the index of visual fatigue (Lambooij et al., 2007). Visual fatigue is closely associated with brightness contrast and stimulus duration. The excessive use of bright or highly saturated colors can have adverse effects on visual perception (Jiang et al., 2011). Therefore, a comprehensive examination of color perception can incorporate the metric of visual comfort through eye-tracking. Luminance contrast is a measure of the difference in brightness between an observed object and its adjacent background within the visual field (Xing et al., 2015). High luminance contrast is associated with enhanced visual perception and reduced fatigue (Tian et al., 2022). Additionally, visual discomfort may change over time and may intensify after prolonged exposure to visual stimuli (Tam et al., 2011). These effects are not as apparent with transient stimuli. Children playing with toys engage in complex tasks that last for certain durations; therefore, short-term stimuli are not suitable for fully exploring children’s perceptions of toy colors.

Second, screen-based experiments involve the presentation of static color images to monitor children’s eye movements and typically consist of discrete trials and repetitions of certain stimuli to capture children’s attention (Yurkovic et al., 2021). Such methods fail to capture the dynamic nature of visual perception and the means by which children engage with their visual environments through active exploration (Slone et al., 2018). In contrast, head-mounted eye-tracking, with its portability and freedom to account for head and body movements, can capture children’s active visual perceptions during free play. This implies that children can choose the stimuli of interest to them at any given time throughout the experiment.

To address the aforementioned questions, this study aimed to use the highest saturated color found in mainstream children’s toys to investigate the presence of a group of colors along the color gradient with higher brightness yet reduced levels of saturation that are more appealing to children. We posit two hypotheses: First, compared with the baseline, when color brightness increases to a certain level, despite a loss of saturation, the color becomes more attractive to children. Second, compared with the baseline, when color saturation loss exceeds a certain threshold, the color loses appeal to children. Our research findings are intended to offer practical guidance on how to design toys that not only capture children’s attention but also ensure visual comfort, thereby unveiling the intricate characteristics of children’s color preferences.

## 2 Materials and methods

### 2.1 Participants

This study collected data through the observation of 23 children: 11 boys and 12 girls. All participants were recruited from the Southeast University Affiliated Kindergarten. However, five children were excluded from the analysis due to insufficient data (defined as having <2 min of usable data). Consequently, a total of 18 children (9 boys and 9 girls) contributed robust eye-tracking data, which provided substantial support for the findings of this study.

We selected children within this specific age group because during the early developmental stages, particularly the preschool phase, the formation of children’s color perceptions and preferences is a complex and critical process that has a significant impact on their subsequent development. To be eligible for inclusion, participants were required to have healthy eyes, normal vision, myopia not exceeding 300 diopters, and the absence of color blindness or deficiency. Prior to the commencement of the experiment, each child completed the Hardy–Rand–Ritter Fourth Edition color vision test, which is designed for preschool children (Xie et al., 2014; Tekavčič Pompe, 2020). Before their participation, researchers provided a comprehensive explanation of the research objectives to all parents and obtained informed consent. Detailed information about all participants is provided in Supplementary Material 1.

### 2.2 Stimuli

Due to the challenges of controlling all three perceptual dimensions of color—hue, chroma, and brightness—in color perception research (Wilms and Oberfeld, 2018), this study primarily focuses on color gradient as the main variable. Hue is categorized into six primary values: red, orange, yellow, green, blue, and purple, which are considered secondary variables and are not the primary focus of discussion (Singh, 2006). Alterations in color gradients lead to changes both in saturation and brightness, making precise stimulus chromaticity specifications essential. Many previous studies on color perception have lacked sufficiently accurate stimulus chromaticity specifications, failed to control saturation and brightness when presenting stimuli of different hues (Kaiser, 1984; Elliot and Maier, 2014), or utilized the non-uniform Munsell color space (Singh, 2006), which does not align with human color perception. To quantify color gradients, we proposed stimuli controlled for chromaticity based on the Commission Internationale de l’Eclairage - Lab (CIELAB) color space (Sharma et al., 2005) and aimed to achieve perceptual uniformity among participants (Robertson, 1990).

We used cubic building blocks (3 cm × 3 cm × 3 cm) as the primary material for the experiment. Each set of building blocks consisted of 24 color-rich blocks, which we carefully selected for the experiment and to which the chosen colors were evenly applied.

Due to the varying ranges of saturation and brightness in different color dye shades, attainment of uniform saturation and brightness levels is unfeasible among dyes of diverse hues. Moreover, the numerical changes in saturation and brightness during the color mixing process exhibit inconsistencies across different hues. Nevertheless, all these hues adhered to the principle of toning by integrating white pigment to heighten brightness while reducing saturation. To enhance the visibility of our experimental results, we aimed to maximize the color differences for each hue. For this purpose, we selected the most highly saturated color achievable within each hue as the baseline for our color gradient. These baseline dyes significantly differed in color from the white dyes typically used in wooden toy production. Hence, we utilized a chroma meter to measure the dyes used in the production of building block toys, including the highest saturated colors: red, orange, yellow, green, blue, and purple, as well as the lowest saturated color, white (Table 1). Through direct measurements, we obtained various colors for the building block toys. Compared with red, orange, yellow, green, blue, and purple, white exhibited higher brightness and lower saturation.

**Table 1:** Color difference between six colors and white.

	Red	Orange	Yellow	Green	Blue	Purple	White
Luminance	48.27	61.90	73.33	46.92	42.60	46.37	95.21
Chroma	56.22	63.62	76.44	56.39	41.81	43.29	0.96
Hue (ab)	24.30	37.20	74.20	149.10	268.70	301.20	222.90

Based on measurements of the baseline dye, we added white dye multiple times to enhance brightness and decrease saturation. This process was divided into several color gradients. To ensure equal perceptual differences between each gradient, we quantified the perceptual difference between two colors using color difference calculations (Commission Internationale de l'Eclairage, 2018). In this study, we used the CIEDE2000 International Commission on Illumination color difference standard (Luo et al., 2001). The color difference gradient was divided based on saturation and brightness, with hue not factored into the calculation (i.e., ∆H = 0). Consequently, we simplified the formula as follows:

$\Delta E_{00}^{*}=\sqrt{{\left(\frac{\Delta L'}{k_{L}S_{L}}\right)}^{2}+{\left(\frac{\Delta C'}{k_{C}S_{C}}\right)}^{2}}$ , $\Delta L'=\Delta L_{2}^{*}-\Delta L_{1}^{*}$ , $\bar{L}=\frac{L_{1}^{*}+L_{2}^{*}}{2}$ , $\bar{C}=\frac{C_{1}^{*}+C_{2}^{*}}{2}$ , $a_{1}^{\prime }=a_{1}^{*}+\frac{a_{1}^{*}}{2}\left(1-\sqrt{\frac{C^{-7}}{C^{-7}+25^{7}}}\right)$ , $a_{2}^{\prime }=a_{2}^{*}+\frac{a_{1}^{*}}{2}\left(1-\sqrt{\frac{C^{-7}}{C^{-7}+25^{7}}}\right)$ , $S_{L}=1+\frac{0.015{\left(\bar{L}-50\right)}^{2}}{\sqrt{20+{\left(\bar{L}-50\right)}^{2}}}$ , $S_{C}=1+0.045C^{-1}$ , where $k_{L},k_{C}$ = 1.

**Figure 1:**
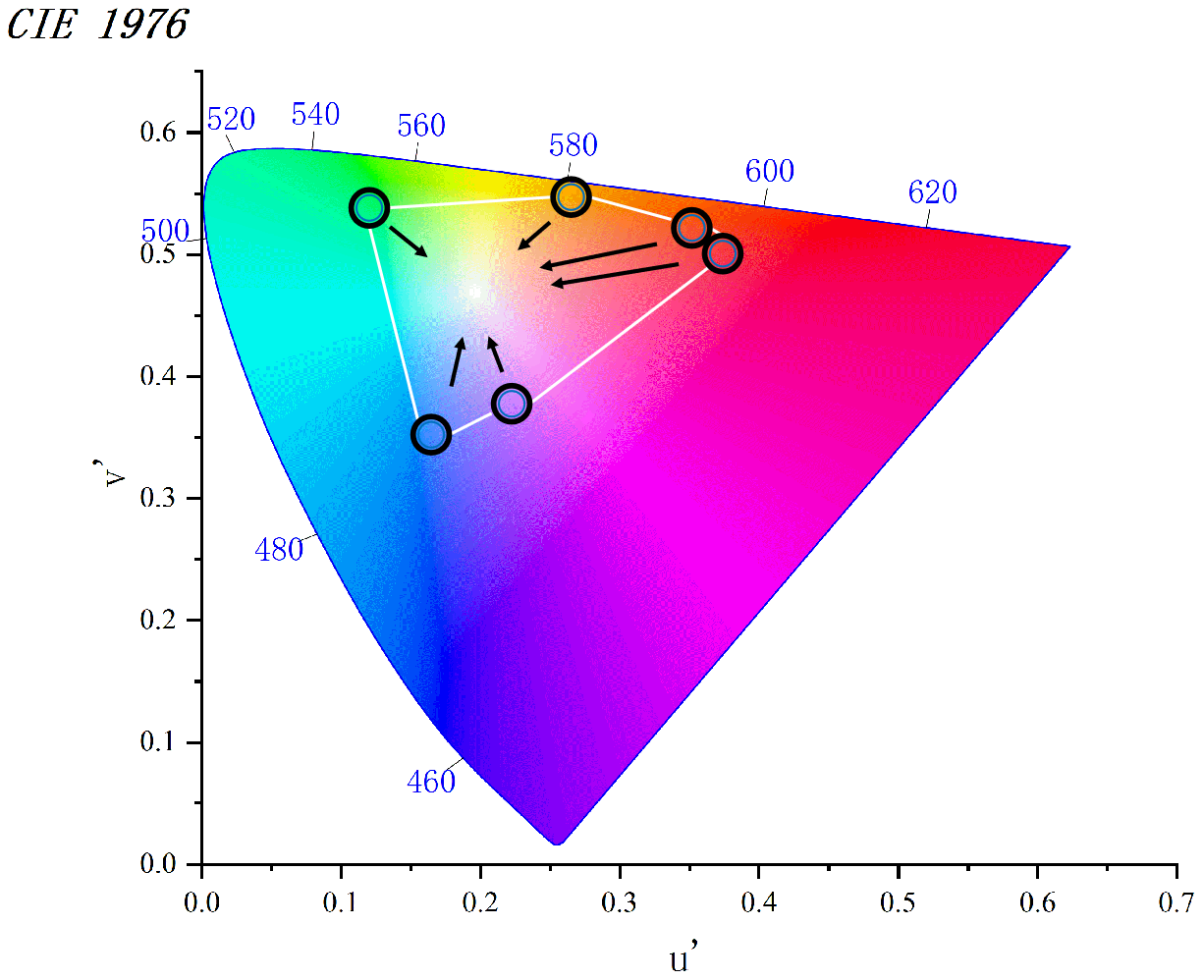
CIE1976 chromaticity diagram.

**Figure 2:**
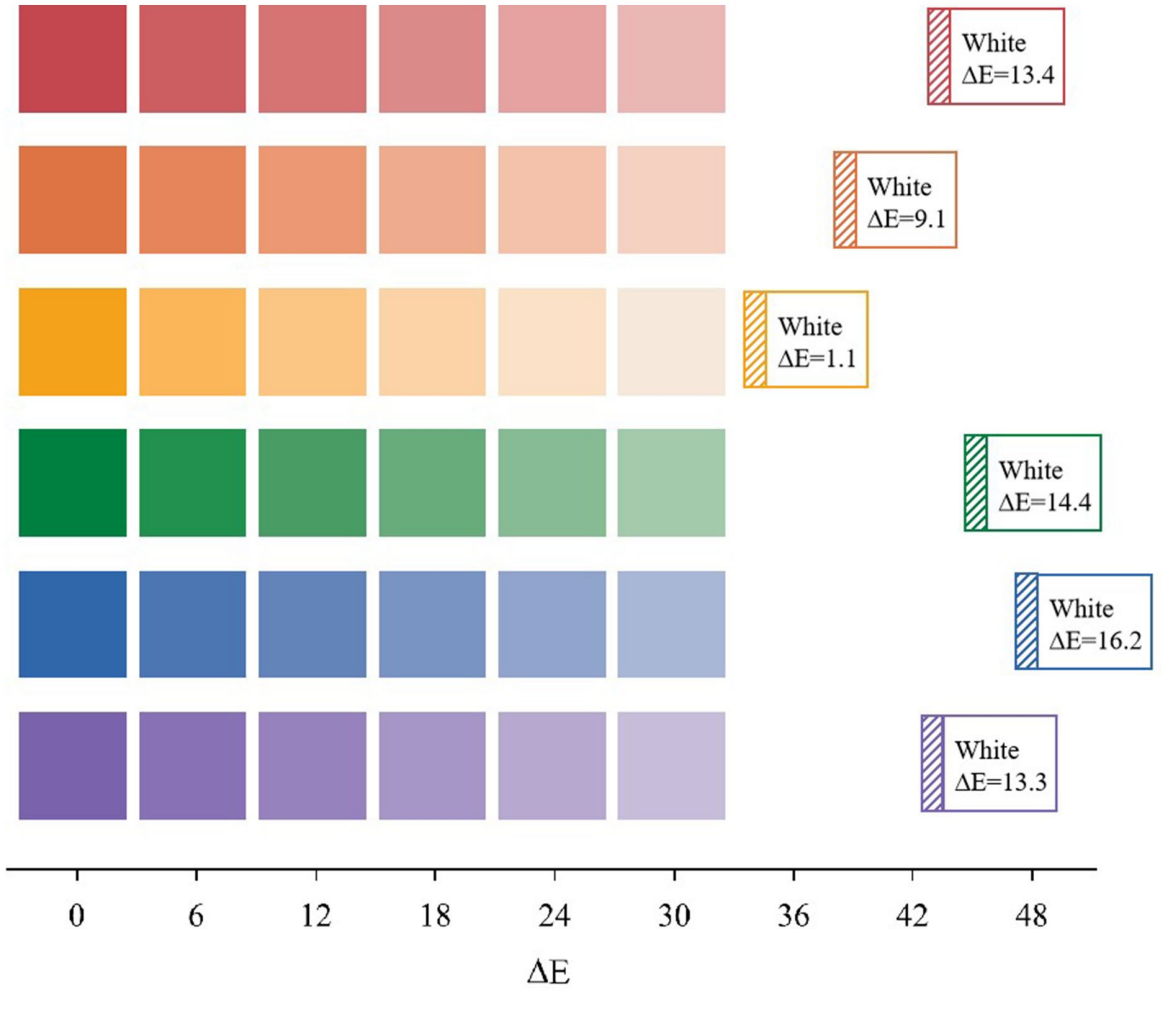
Color gradient map.

As shown in Figure 1, we took the six measured colors as reference points and found that each color occupies a different position in the color space. To ensure that the degree of deviation of each color toward white was equal, we specified the color gradient difference. After performing the calculation, we determined that yellow had the smallest color difference from white, with ΔE = 31.121. Therefore, we set the gradient color difference ΔE to 6 in the experiment. This way, each group of colors was divided into six gradients, as shown in Figure 2.

The experiment materials were pre-adjusted based on the calculated colors, and a colorimeter was used to measure the pre-adjusted colors. During this process, we continuously fine-tuned the pigment colors until their color deviation from the ideal saturation was <3%.

### 2.3 Experimental environment

The experimental area was situated in a spacious and quiet indoor environment, furnished with tables and chairs for participants to comfortably engage in the experiments. To mitigate metamerism (Akbarinia and Gegenfurtner, 2018) and eliminate the influence of ambient light and background on participants’ color perception (Brainard and Wandell, 1992), we maintained uniform environmental lighting and background.

**Figure 3:**
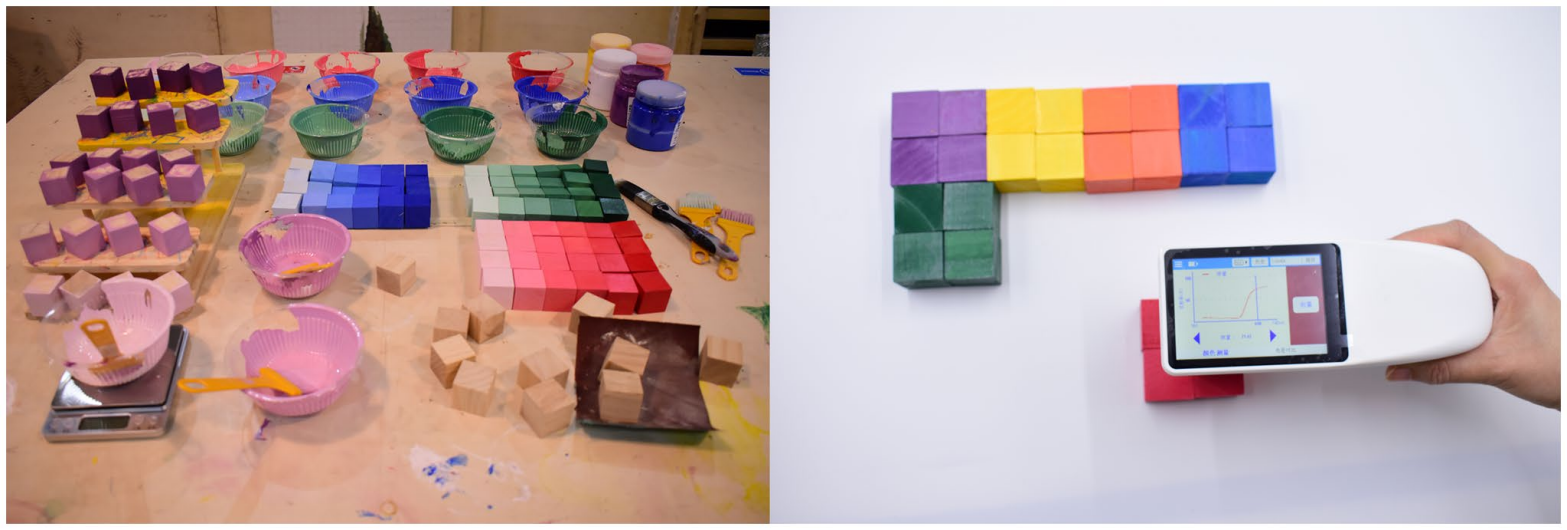
Preparation of experimental materials.

To achieve these objectives, we carried out a carefully designed experimental setup, with a key component being a large lightproof enclosure measuring 60 cm × 60 cm × 60 cm. Each of the enclosure’s five sides was covered with blackout curtains that effectively blocked external light, ensuring that the internal environment remained unaffected by external lighting conditions. Inside the enclosure, an adjustable white light source was positioned on top, surrounding the interior to provide uniform illumination from all angles. Additionally, a pure white plastic board was installed as a background within the enclosure to ensure a consistent color background environment. Throughout the experiment, a photometer was used to monitor and maintain consistent illuminance inside the enclosure (Figure 3).

**Figure 4:**
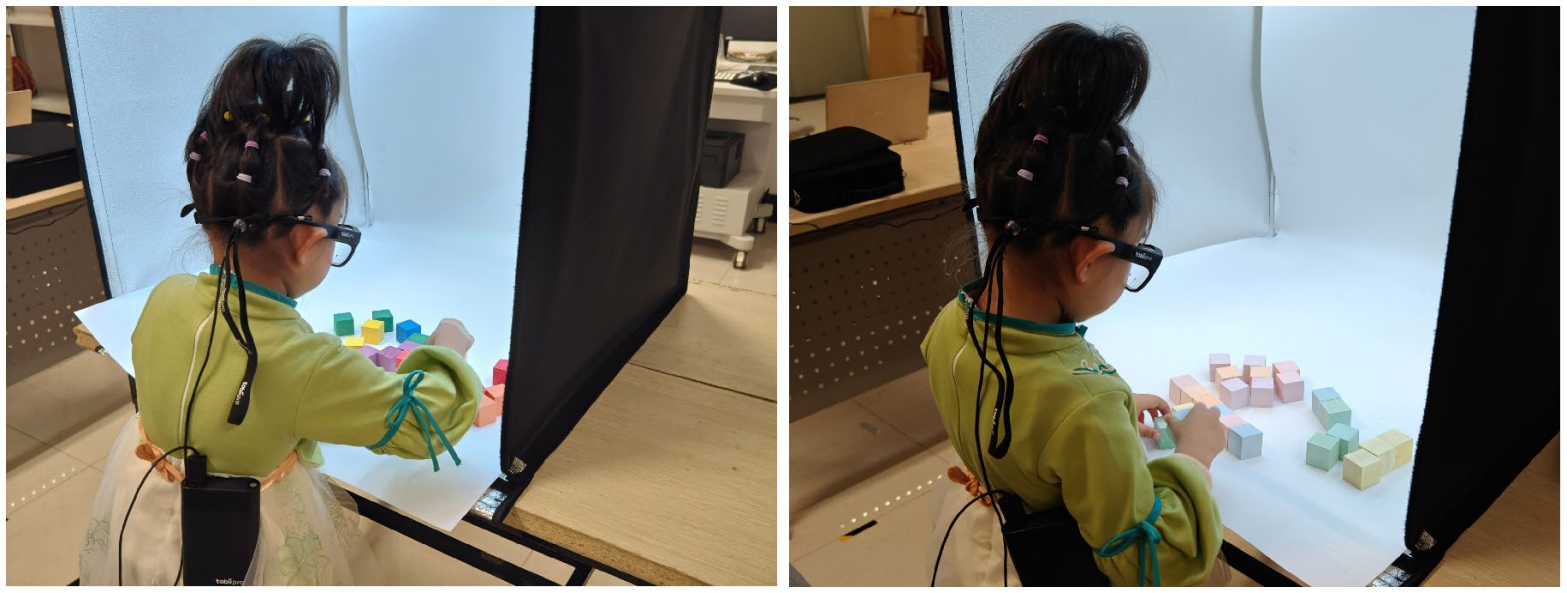
Experimental setup and environment.

Simultaneously, to eliminate interference from external light and debris, each participant’s field of vision was confined within the enclosure. This setup ensured the consistency of the experimental environment, thereby enhancing the reliability and comparability of the experiments. The experimental facility is depicted in Figure 4.

### 2.4 Procedure

The experimenter invited the children to the Key Laboratory of Child Development and Learning Science (Southeast University), Ministry of Education. The children sat in front of the experimental box, and the experimenter wore the Tobii Glass 2 head-mounted eye tracker for the children. Specifically tailored Tobii glasses nose rests were used for child participants to align their eyes with the eye-tracking area. The experimenters also adjusted and secured the glasses using a strap to ensure that the Tobii glasses remained in place and to prevent any slippage during the course of the experiment.

Prior to starting the experiment, a calibration procedure was mandatory, and the experiment could only officially begin after successful calibration. Throughout the experiment, no instructions or requests were imposed by the experimenter on the children. Instead, they were allowed to play with the toys freely within the specified time.

**Figure 5:**
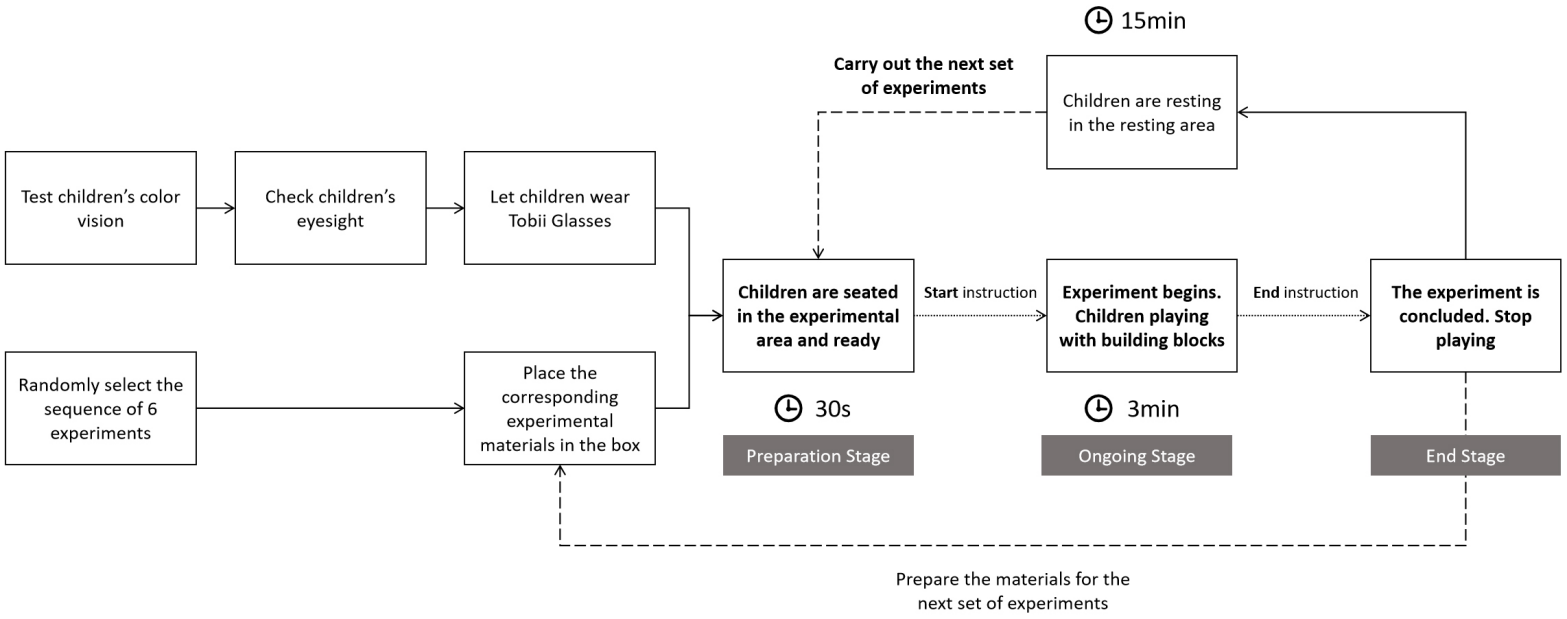
Experimental procedure.

In this experiment, six sets of building blocks (S1, S2, S3, S4, S5, and S6) were prepared. Each child participant played with the six sets of building blocks in a sequential manner, resulting in a total of six color experiments. Considering the characteristics of children, their attention span, and comfort, each experimental session lasted for 3 min. In total, 108 experiments were conducted. To eliminate sequence effects in the experiments, the order of participation in the six experimental sets was randomized for each participant, ensuring that no fixed order was established, thereby avoiding any influence on the experimental results. Furthermore, a 20-min break was provided between each experimental set to mitigate fatigue effects and ensure that the performance of each set was not affected by the preceding set. The entire experimental procedure is illustrated in Figure 5.

### 2.5 Eye movement indicators

#### 2.5.1 Color of interest

Areas of interest (AOIs) are typically carried out in research to investigate specific visual attention patterns and attention distribution (Borys and Plechawska-Wójcik, 2017). In the context of human–object interaction, AOIs are used to quantify users’ attention to specific regions while performing particular tasks (Poole and Ball, 2006). AOIs serve as direct evidence of the attractiveness of certain elements in various studies. For example, AOI analysis has been used to assess the relative importance of the mouth and teeth in facial attractiveness (Hickman et al., 2010), evaluate the attention-grabbing effect of food labels as AOIs for consumers (Ares et al., 2013), and analyze AOIs in recommendation interfaces to enhance user engagement.

Fixation refers to the process of maintaining the central foveal vision on a target for a certain duration to gather sufficient visual image details (Just and Carpenter, 1976). Studies have shown that fixation time (FT) can reflect each participant’s familiarity and level of attention to specific information (Christianson et al., 1991). AOIs are obtained by calculating the FT within defined and fixed regions (Jacob, 1995). However, since our experiment involved capturing the visual information seen by participants through the front camera of the head-mounted eye tracker, instead of using a fixed screen, conventional AOI methods could not be applied. In a dynamic visual environment, flexible AOIs must be determined. Gomolka et al. (2020) performed the Tobii Glasses Pro head-mounted eye tracker and developed a specific method to accumulate the FT for each dial, resulting in flexible AOI metrics.

In our experiment, we used the concept of colors of interest (COIs) as a replacement for traditional AOI metrics. COI can be understood as the proportion of FT on colored building blocks relative to the total time in a dynamic visual context. In terms of calculation, we considered the display area of each building block entity in the captured images as flexible AOIs. By merging the flexible AOIs for all building blocks, we obtained the COI for each specific color gradient. Additionally, summing the flexible AOIs for building blocks of the same color allowed us to analyze the interest distribution for different colors within the same color gradient.

**Figure 6:**
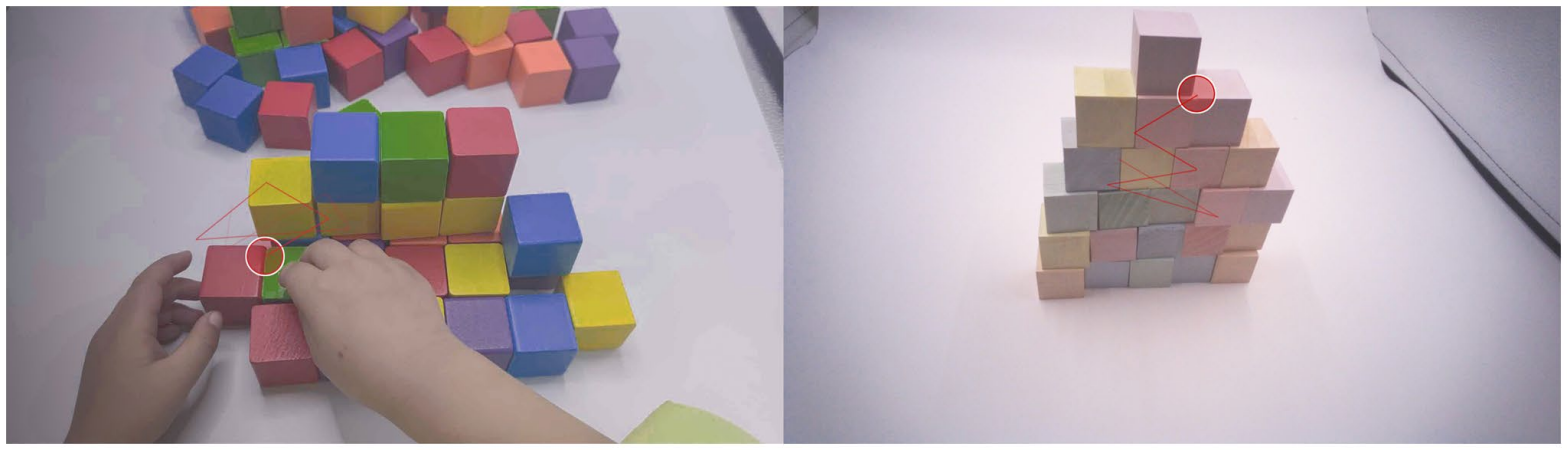
Visual field images and gaze point coordinates.

Tobii Pro Glasses 2 recorded the participants’ visual field images and gaze point coordinates during the experiment. The recorded video frames each had a size of 1,920 pixels × 1,080 pixels, a frame rate of 25.02 frames per second, and a data rate of 4,992 kbps. The gaze coordinates contained timestamps and coordinate points within the range of (0, 0) to (1,920, 1,080), where the top-left corner of the video was considered (0, 0), with the x-axis extending to the right and the y-axis extending downward. Aligning the video timestamps with the gaze point timestamps allowed us to determine the targets on which the participants fixated in their fields of vision, as illustrated in Figure 6.

To obtain the flexible AOIs for each building block, identification of the fixation points was necessary. Potential fixation points within the enclosure included colored building blocks, the white background, and the participant’s hands. We observed substantial differences in the color compositions of these three types of fixation points. The white background and colored building blocks exhibited significant contrast, while the color of human skin in the video had lower saturation and brightness. Therefore, we could filter out fixation points related to hands and the background by setting saturation and brightness thresholds. The colored building blocks had both bright and dark sides, resulting in varying color information in the video. However, different faces of the same color building block had small differences in hue in the video, while the hue differences between different color building blocks were significant. Thus, we could determine the color attribution of the building blocks based on each block’s hue information.

In practice, we developed a program to identify the color block in which the fixation point coordinates were located. We extracted the color information from the surrounding 3 cm × 3 cm color blocks and then calculated the mean hue, chroma, and luminance values of the nine color blocks to eliminate the influence of video artifacts and defects. The resulting hue–chroma–luminance (HCL) values of the processed color blocks represented the color information corresponding to the participant’s fixation point at that time. We used these colors in a filter, as shown in Table 2, to determine whether the participant fixated on a colored building block and to identify the color of the building block.

**Table 2:** Judgment range of color.

	Red	Orange	Yellow	Green	Blue	Purple
Hue	312,360 or 0,20	20,50	50,68	68,170	170,248	248,312
Chroma	20,100					
Luminance	20,100					

Hue is from 0 to 360; chroma is from 0 to 100; luminance is from 0 to 100.

The colors captured in the video by the head-mounted eye tracker could exhibit slight distortions. However, a significant difference was identified between the color information of colored building blocks and the background or human skin in the video. By setting the filter to chroma >20 and luminance >20, we could effectively filter out fixation points related to the background and the participant’s hands. Subsequently, we categorized the filtered data based on the hue values within specified ranges to determine the color. The video color distortion did not exceed the redundancy set by the filter.

The duration of each experiment was T; the sampling rate of Tobii Glasses Pro 2 was $f_{s}$ =100 Hz; the interval between each judgment was *t* = 10 ms; the interval of each video frame was $t_{F}=40\;\mathrm{ms}$ ; and each frame was judged four times. The three types of eye movements were fixation, saccade, and unclassified (Groner et al., 2021). The total number of judgments was N, the number of fixations was $N_{F}$ , number of sweeps was $N_{S}$ , and unrecognized numbers was $N_{U}$ , $N=N_{F}+N_{S}+N_{U}$ . Colors were denoted as follows: red $\left(n_{R}\right)\text{,}$ orange $(n_{O}$ ), yellow ( $n_{Y}$ ), green ( $n_{G}$ ), blue ( $n_{B}$ ), and purple ( $n_{P}$ ). The formula used for fixations was as follows: $N_{F}$ = $n_{R}+n_{O}+n_{Y}+n_{G}+n_{B}+n_{P}+n_{N}$ , where $n_{N}$ is an invalid judgment. Thus, the total FT for each experiment was $T_{FT}$ = $N_{F}$ t, and the calculation formula was as follows:

$$COI=\frac{\left(N_{F}-n_{N}\right)t}{T-N_{U}\times t}\times 100\%$$

#### 2.5.2 Pupillary unrest index

The pupillary unrest index (PUI) was defined as the cumulative variation in pupil size based on the mean values of continuous data sequences (Lüdtke et al., 1998). This parameter can objectively measure the degree of unstable pupil movement (Warga et al., 2009) and serves as an important metric for assessing visual fatigue (Nakayama, 2006). PUI is negatively correlated with the level of visual comfort (Tyukhova and Waters, 2019), and a smaller PUI value is associated with a more comfortable visual experience and a greater attractiveness to visual attention. According to the calculation standard of $\frac{25}{16}$ Hz, when the sampling rate is $f_{s}$ =100 Hz, 64 consecutive pupil diameters are recorded per second, and $d_{i}$ is the average value of 64 continuous pupil diameters. Therefore, the formula for PUI was:

$$PUI=\frac{1}{\left(N-64\right)\Delta t}\cdot \overset{\frac{N}{64}}{\underset{i=2}{\sum }}\left|d_{i}-d_{i-1}\right|$$

#### 2.5.3 Saccade rate

Saccades are a process in which the foveal visual field discontinuously moves from one location to another quickly (Lai et al., 2013). The number of saccades ( $N_{S})$ reflects the degree of mental and muscle activity or fatigue of an individual to a certain degree; however, limitations exist owing to the large initial physiological differences of individuals. In this experiment, the saccade parameter and saccade rate (SR) of the ratio nature were chosen instead of $N_{S}$ , for judgment. The formula was as follows:

$$SR=\frac{N_{s}}{N}\times 100\%$$

### 2.6 Data analysis

Regarding the COI, PUI, and SR metrics, we initially assessed the normality of each metric using the Shapiro–Wilk test. Once the data for each group satisfied the normality assumption, we conducted a test for homogeneity of variances using Bartlett’s test. For cases in which the normality assumption was violated, we performed Levene’s test for variance homogeneity.

Specifically, for the PUI and COI metrics, the results of the Shapiro–Wilk tests indicated that data from all groups satisfied the normality assumption. Bartlett’s test was subsequently carried out to test the homogeneity of variance. For the SR metric, the normality assumption was found to be violated for some groups, as indicated by the results of the Shapiro–Wilk test. Consequently, we performed Levene’s test for variance homogeneity. For all three metrics, the assumption of variance homogeneity was satisfied.

To compare the differences in these metrics across different groups, for COI and PUI, we performed one-way analysis of variance (ANOVA) since all assumptions were met. Post-hoc analysis was carried out using Tukey’s honestly significant difference (HSD) tests. For SR, the normality assumption was violated, and we performed a non-parametric analysis by applying the aligned ranks transformation ANOVA method (Elkin et al., 2021).

## 3 Results

### 3.1 Color gradient physiological data

This study involved the measurement of children’s physiological indicators under different color gradients. COI results showed that S5 had the highest COI value, with an increasing trend in COI values from S1 to S5, an elevation in brightness, and a reduction in saturation. However, a significant drop in COI values was observed from S5 to S6, despite the continued increase in brightness and further reduction in saturation. Furthermore, PUI results revealed that the PUI value was the lowest for S5, with a decreasing trend in PUI values from S1 to S5, accompanied by increased brightness and reduced saturation. However, from S5 to S6, PUI values increased, despite the continued increase in brightness and reduction in saturation. In addition, SR results indicated that S6 had the highest SR value, while S2 had the lowest SR value, with no apparent pattern observed among the remaining gradients.

ANOVA for COI showed significant intergroup differences (F[5, 102] = 32.03, *p* < 0.001, η ^2^ = 0.610). Post-hoc comparisons indicated that the baseline group S1 (∆E = 0) exhibited statistically significant differences (*p* < 0.05) when compared with S3 (∆E = 12), S4 (∆E = 18), S5 (∆E = 24), and S6 (∆E = 30), with the exception of the adjacent pairs S5 (∆E = 24) and S6 (∆E = 30; *p* > 0.05).

Combining the mean analysis, the mean values increased continuously from S1 (M = 0.508) to S5 (M = 0.743), indicating that children’s color preferences rise with increasing color brightness (S1–S5), reaching their peak at S5 (M = 0.743). This result supports our initial hypothesis (H1), suggesting that children have a preference for brighter colors within a certain brightness threshold.

**Figure 7:**
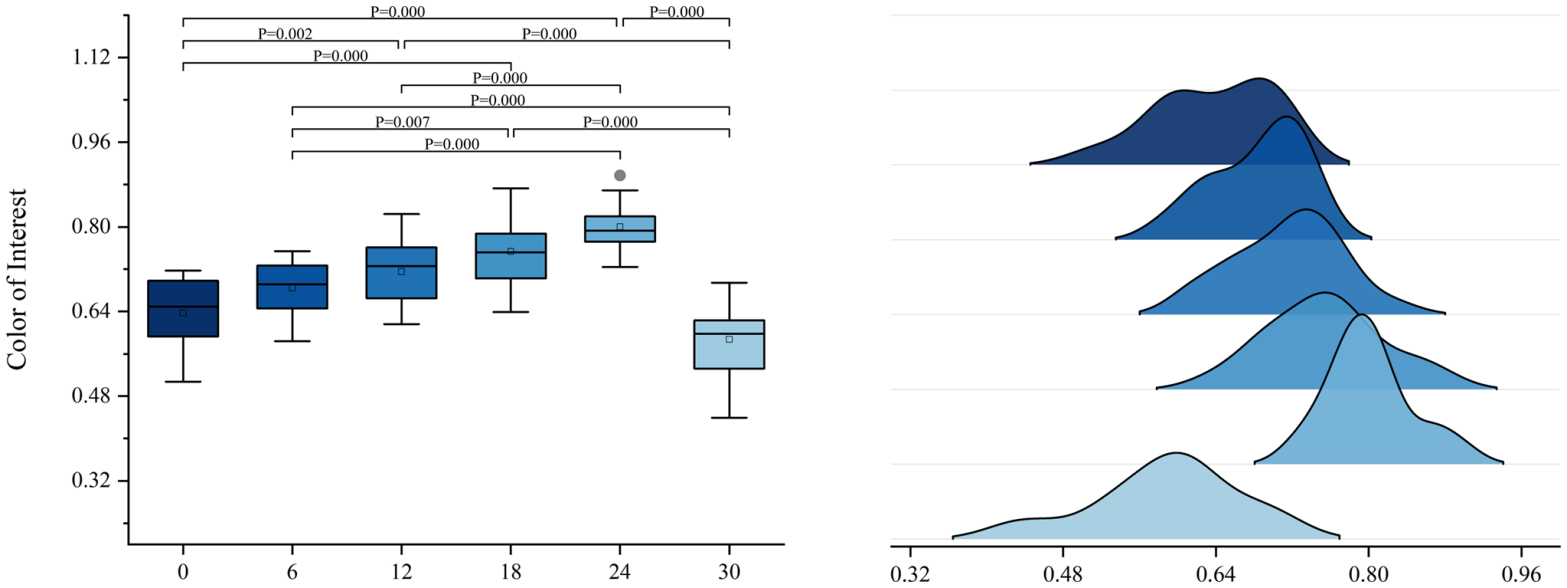
Analysis of variance outcomes of color of interest.

However, a sharp decline was identified in mean values from S5 (M = 0.743) to S6 (M = 0.440; *p* < 0.001), and the mean value at S6 (M = 0.440) was lower than the baseline value at S1 (M = 0.508). This finding indicates that children’s attraction to color diminishes markedly when brightness is further increased and saturation is further reduced. This further suggests that beyond a certain brightness threshold, the appeal of color to children does not increase proportionally, and, at this point, the negative impact of reduced saturation outweighs the positive effect of increased brightness. This result supports our second hypothesis (H2; Figure 7).

ANOVA for PUI revealed statistically significant group differences (F[5, 102] = 2.992, *p* = 0.015, η ^2^ = 0.128). Post-hoc tests indicated a significant difference (p < 0.05) between S5 (∆E = 24) and S6 (∆E = 30), while no significant differences (p > 0.05) were found among S1 (∆E = 0), S2 (∆E = 6), S3 (∆E = 12), S4 (∆E = 18), and S5 (∆E = 24).

**Figure 8:**
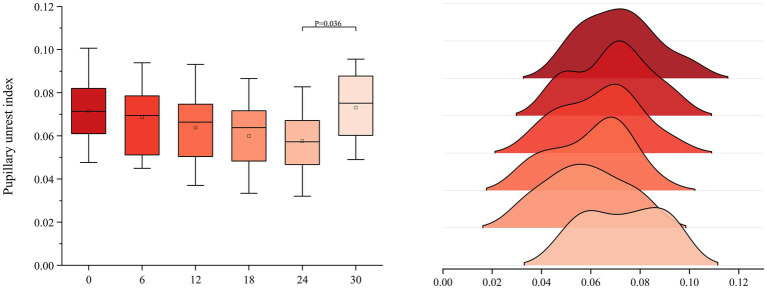
Analysis of variance outcomes of pupillary unrest index.

Considering the mean analysis, the mean values decreased continuously from S1 (M = 0.082) to S5 (M = 0.067) and then increased from S5 (M = 0.067) to S6 (M = 0.091). Notably, the mean value of S6 (M = 0.091) exceeded that of S1 (M = 0.082). This indicates that within the range of saturation from S1 (∆E = 0) to S5 (∆E = 24), children’s comfort during play slightly increased as saturation decreased and brightness increased, although this increase was not significant. However, from S5 (∆E = 24) to S6 (∆E = 30), the visual comfort of the toy colors decreased as saturation further decreased, implying that children experienced discomfort during play (Figure 8).

**Figure 9:**
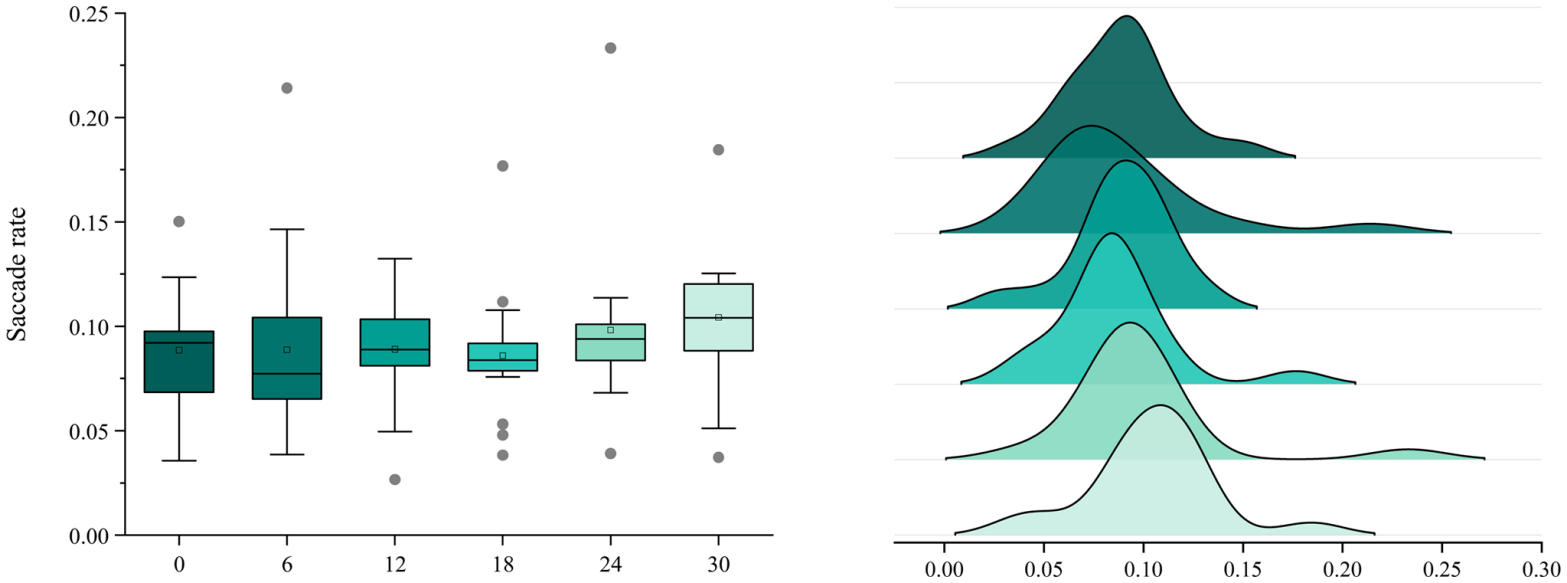
Analysis of variance outcomes of saccade rate.

Regarding SRs, ANOVA indicated no statistically significant differences among the levels (F[5,102] = 2.240, *p* = 0.056). The mean values for each level were as follows: S1 (M = 0.150), S2 (M = 0.112), S3 (M = 0.132), S4 (M = 0.101), S5 (M = 0.233), and S6 (M = 0.088). No significant differences were identified in mean values among the compared levels, and SR values did not differ significantly across different color gradients (Figure 9).

### 3.2 Color distribution of COI

**Figure 10:**
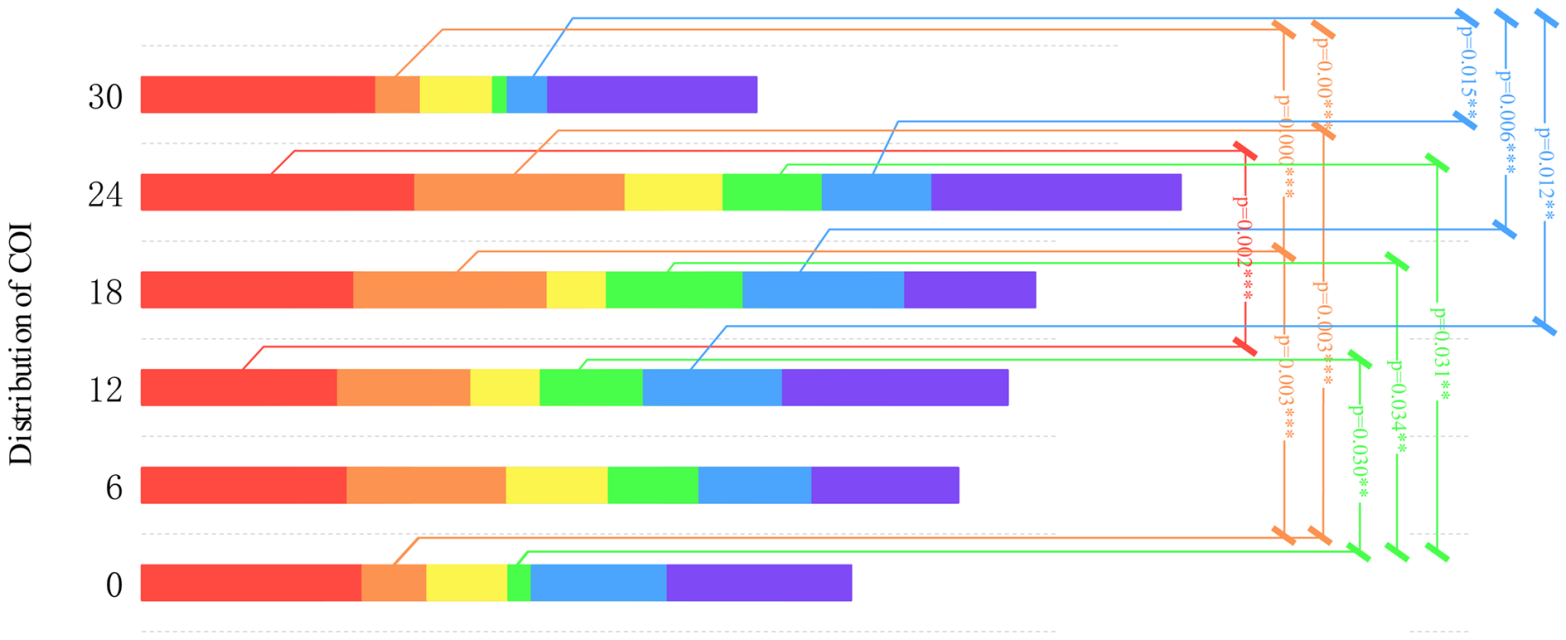
Color distribution of color of interest.

Differences were observed in the specific color allocations of COI under different color gradients. ANOVA was conducted on the COI color allocation for different color gradient groups, as shown in Figure 10.

Differences in red COI among different color gradients were statistically significant (F[5,102] = 4.199, *p* = 0.005, η ^2^ = 0.412). Post-hoc tests indicated a significant difference (*p* = 0.002) between S3 (∆E = 12) and S5 (∆E = 24). Combining the mean analysis, the allocation ratio of red color was observed to be the highest and remained relatively stable across different gradients. This implies that the color red is significantly attractive across all the gradients.

Similarly, differences in orange COI were statistically significant (F[5,102] = 8.613, *p* < 0.001, η ^2^ = 0.589), with significant differences (*p* < 0.05) observed between S1 (∆E = 0) and S4 (∆E = 18), S1 (∆E = 0) and S5 (∆E = 24), S4 (∆E = 18) and S6 (∆E = 30), and S5 (∆E = 24) and S6 (∆E = 30). These findings indicated that variations in the color orange across different gradients significantly influenced children’s preferences.

Differences in yellow COI were not statistically significant (F[5,102] = 2.224, *p* = 0.078), with no significant differences in the overall mean, suggesting that children’s preference for yellow remained relatively consistent across various gradients.

Differences in green COI were statistically significant (F[5, 102] = 3.855, *p* = 0.008, η ^2^ = 0.391), with significant distinctions observed between S1 (∆E = 0) and S3 (∆E = 12), S1 (∆E = 0) and S4 (∆E = 18), and S1 (∆E = 0) and S4 (∆E = 18). These findings implied that, in specific circumstances, children’s preferences for the color green may vary. Mean analysis indicated that the allocation of the color green in S1 (M = 1.969) was the lowest, while that in S3 (∆E = 12), S4 (∆E = 18), and S5 (∆E = 24) had higher mean values. In situations with higher saturation, children’s preference for this specific color may decrease.

Differences in blue COI were statistically significant (F[5,102] = 4.609, *p* = 0.003, η ^2^ = 0.434), with significant differences observed between S3 (∆E = 12) and S6 (∆E = 30), S4 (∆E = 18) and S6 (∆E = 30), and S5 (∆E = 24) and S6 (∆E = 30). The mean analysis further emphasized the significant variations in the attractiveness levels of the color blue across these gradients, especially with S6 (M = 4.542) showing relatively lower attractiveness compared with other gradients.

Differences in purple COI were not statistically significant (F[5,102] = 1.631, *p* = 0.182). This finding suggests that the color purple can maintain a relatively consistent level of attractiveness across various gradients.

## 4 Discussion

### 4.1 Expression of color gradient

More vibrant and saturated colors are generally preferred by individuals (Guilford, 1934; Smets, 1982). Guilford proposed that high-brightness colors are more popular than low-brightness colors, and saturated colors are also more favored than unsaturated colors. However, in real production, high saturation and high brightness unfortunately cannot coexist. Within a collection of color gradients, a trade-off relationship exists between saturation and brightness; as saturation gradually decreases, brightness increases. Smets (1982) rated the contributions of these two-color attributes to preference scores, with saturation accounting for 88% of the variance and brightness for 12%. Thus, preferences for different color gradients may exhibit differences influenced by these two attributes. The imbalance in the relative contributions of saturation and brightness necessitates careful balancing and adjustment of these two attributes in actual product design to meet the visual preference needs of children.

Based on the experimental results, we further confirm our first hypothesis, which suggested that the existence of a color gradient (S5) with higher brightness but lower saturation is most preferred by children. The findings indicate that, within a certain range, the increase in visual attractiveness due to brightness enhancement between color gradients outweighs the loss in visual attractiveness caused by reduced saturation. However, the S6 gradient was the least favored by children, indicating that when saturation decreased to a certain point, the visual attractiveness loss due to saturation reduction in the S5–S6 gradient far exceeded the increment resulting from increased brightness. As a result, children’s visual attractiveness rapidly declined. Additionally, the S1 color blocks had the lowest brightness, although they exhibited the highest saturation. Their appeal to children was only marginally better than that of the S6 color blocks.

### 4.2 Visual fatigue under brightness contrast

In the context of a light background, Xie et al. (2021) proposed that higher brightness contrast is beneficial for reducing visual discomfort. Additionally, high-brightness contrast can lead to visual excitement (Benedetto et al., 2014), while excessively low-brightness contrast can impair eye accommodation and make focusing attention difficult (Schwartz, 2008). Ou et al. (2015) reported that young people experience the highest visual comfort when brightness contrast is moderate rather than extremely high; this conclusion was corroborated by our study. Since the experimental background was white, the brightness contrast between S1–S6 and the white background gradually decreased. PUI values for S1–S5 decreased as brightness contrast decreased, indicating that moderate brightness contrast led to comfort for children. However, as the color gradients approached a similarity to the white background, PUI values sharply increased. This suggests that excessively low-brightness contrast during play tasks may have made it difficult for children to distinguish between the background and the toy blocks, resulting in lower PUI due to their inability to concentrate effectively. Therefore, in the color design of toys, a balance between brightness and contrast is essential, ensuring that brightness contrast is neither too high nor too low. This will aid children in perceiving and distinguishing toy colors more effectively, thereby enhancing their visual comfort.

### 4.3 Children’s preferences for different hues in a color set

Figure 10 provides a comprehensive analysis, shedding light not only on children’s preferences for diverse color gradients but also enabling a deeper exploration into their preferences within the same color set. Across all color sets, our findings indicated higher attractiveness for red and purple, while yellow consistently showed lower attractiveness. Notably, significant differences were identified in the perceptions of orange, green, and blue between S1 and S6 (*p* < 0.05). This phenomenon invites two potential explanations.

First, the differences in children’s perceptions of color brightness thresholds suggest unique individual preferences. Variations in the initial brightness and saturation levels of each color starting point (S1) could result in orange, green, and blue surpassing their specific brightness thresholds by the time they reached the S6 level. This excessive brightness might reduce children’s attention, compared with that of the S1 baseline. Conversely, the brightness of red or purple at the S6 level may not have exceeded their respective specific thresholds, thereby sustaining heightened attention.

Second, children may have exhibited stable preferences for specific colors. Prior research, such as that conducted by Wilson (1966), noted red as one of the most favored colors. Hence, children might have an innate inclination toward red and purple, irrespective of whether their brightness surpasses a certain threshold. Conversely, an inherent disinclination might exist among children toward colors such as orange, green, and blue, regardless of whether these colors exceed a certain threshold of brightness.

### 4.4 Two possibilities for the influencing factors of “cold-warm” color preference

Figure 10 displays the proportion of warm colors (red, orange, and yellow) in the color COIs for different color gradients, which were 60.14, 57.29, 52.04, 58.40, 58.85, and 60.43%, respectively. However, this result does not confirm a preference for warm color hues among children. Several studies have reported that preferences for ‘warm-cool’ colors are primarily influenced by hue rather than saturation and brightness (Mogensen and English, 1926; Newhall, 1941). Conversely, other studies have reported the opposite conclusion, suggesting that ‘warm-cool’ preferences are mainly determined by saturation, with hue having a less significant impact (Gao and Xin, 2006). In our experiment, due to variations in saturation and brightness of the dyes under different hues, the saturation and brightness of each color gradient are closely related to the baseline color’s saturation and brightness. Specifically, warm color hues in the same color gradient have higher saturation and brightness compared with cooler color hues. Therefore, conclusive evidence has not been determined regarding the influence of hues. In future research focusing on preferences for ‘warm-cool’ colors, controlling both saturation and brightness variables may be necessary to gain a better understanding of such preferences. Therefore, this result does not conclusively demonstrate a preference for hue. Future research can eliminate the influence of these factors on hue preference by exerting more precise control over saturation and brightness as two variables.

## 5 Limitations

While this study provided valuable findings, several noteworthy limitations must be acknowledged. First, our study considered only six different color gradients, and the granularity of the experimental color gradients was relatively coarse, which may limit our comprehensive understanding of the complex relationship between brightness and saturation. For future research, we recommend delineating finer color gradients to obtain more precise saturation and brightness thresholds, ensuring a vivid and bright visual effect. Additionally, this study had a limited sample size, and the sample was primarily concentrated within a specific age range of children. To some extent, this restricted the study’s generalizability and external validity. Future research can expand the sample size and include children from diverse age groups and cultural backgrounds to achieve more representative results.

## Data availability statement

The raw data supporting the conclusions of this article will be made available by the authors, without undue reservation.

## Ethics statement

The studies involving humans were approved by IEC for Clinical Research of Zhongda Hospital, Affiliated to Southeast University. The studies were conducted in accordance with the local legislation and institutional requirements. Written informed consent for participation in this study was provided by the participants’ legal guardians/next of kin. Written informed consent was obtained from the individual(s), and minor(s)' legal guardian/next of kin, for the publication of any potentially identifiable images or data included in this article.

## Author contributions

ZC: conceptualization, methodology, validation, investigation, data curation, writing-original draft, and visualization. TY: methodology, software, validation, formal analysis, data curation, writing-original draft, writing-review and editing, and visualization. YC: software, formal analysis, data curation, methodology, visualization, writing-original draft, and writing-review and editing. TC: investigation, resources, supervision, and funding acquisition. SC: supervision, project administration, validation, funding acquisition, resources, writing-review and editing. All authors contributed to the article and approved the submitted version.
