# Supplementary material for: Using head-mounted eye trackers to explore children’s color preferences and perceptions of toys with different color gradients
Source: Front Psychol. 2023 Dec 21;14:1205213. doi: 10.3389/fpsyg.2023.1205213 (PMC10771309; doi:10.3389/fpsyg.2023.1205213)
Supplement: Supplementary file 1 [file Table_1.DOCX]

Supplementary Material 1

1.Participant Information Table

| ID | Age | Gender | Favorite Color |
| --- | --- | --- | --- |
| 1 | 5 | girl | Pink |
| 2 | 6 | boy | Blue |
| 3 | 5 | boy | Yellow |
| 4 | 5 | boy | Blue |
| 5 | 5 | girl | Pink |
| 6 | 4 | boy | Blue |
| 7 | 6 | boy | Orange |
| 8 | 6 | girl | Purple |
| 9 | 4 | girl | Red |
| e | 6 | boy | Green |
| 11 | 7 | girl | Orange |
| 12 | 5 | girl | Pink |
| 13 | 5 | boy | Orange |
| 14 | 5 | girl | Pink |
| 15 | 6 | boy | Blue |
| 16 | 4 | girl | Purple |
| 17 | 5 | girl | Blue |
| 18 | 5 | boy | Green |
| 19 | 4 | girl | Pink |
| 29 | 4 | boy | Blue |
| 21 | 6 | boy | Green |
| 22 | 6 | girl | Blue |
| 23 | 5 | girl | Red |
